# EZH2-mediated epigenetic suppression of lncRNA PCAT18 predicts a poor prognosis and regulates the expression of p16 by interacting with miR-570a-3p in gastric cancer

**DOI:** 10.7150/jca.63415

**Published:** 2021-10-17

**Authors:** Liangjun Zhu, Chongguo Zhang, Jiao Xue, Xuezhi He, Dandan Yin, Qingqing Zhu, Yongqian Shu, Wei De

**Affiliations:** 1 Department of Oncology, First Affiliated Hospital of Nanjing Medical University, Nanjing, Jiangsu, 210000, PR China.; 2 Department of Radiation Oncology, First Affiliated Hospital of Soochow University, Suzhou, Jiangsu, 215006, PR China.; 3 Department of Biochemistry and Molecular Biology, Nanjing Medical University, Nanjing, Jiangsu, 210000, PR China.; 4 Clinical Research Center, The Second Hospital of Nanjing, Nanjing University of Chinese Medicine. Zhong Fu Road, Gulou District, Nanjing, Jiangsu 210003, PR China.; 5 Department of Respiratory Medicine, First Affiliated Hospital of Soochow University, Suzhou, Jiangsu, 215006, PR China.

**Keywords:** EZH2, PCAT18, long noncoding RNA, miRNA, p16, gastric cancer

## Abstract

It was recently demonstrated that long noncoding RNAs (lncRNAs) have key regulation functions in the biology of human cancer. The current study aimed to determine the expression, clinicopathological characteristics and functional roles of lncRNA PCAT18 in gastric cancer (GC).

By analysis of (Gene Expression Omnibus) GEO and TCGA data, following experimental verification, we identified the function role and molecular mechanism of PCAT18 in tumorigenesis of GC. We discovered that PCAT18 is significantly decreased in paired GC tissues and correlates with a poor outcome. Mechanistic studies found that suppression of the expression of EZH2 could prevent its binding to the PCAT18's promoter region and decrease H3K27's trimethylation modification. In addition, PCAT18 could adjust cell proliferation of GC *in vitro* as well as *in vivo*. Further mechanism research revealed that PCAT18 could regulate the expression of p16 by interacting with miR-570a-3p, thus inhibiting cell proliferation of GC. Our results have shown that the histone modification-mediated epigenetic suppression of PCAT18 and its essential role of PCAT18 in GC oncogenesis, which could provide a theoretical basis for GC therapy.

## Introduction

In low- and middle-income countries gastric cancer (GC) is among the main causes of cancer mortality 1, 2, with a 5-year survival of under 30%. The molecular mechanisms related to the tumorigenesis of GC is complicated. Therefore, to enhance GC's treatment and diagnosis, a greater comprehension of molecular mechanisms is needed.

Previous research indicated that genes that encode proteins take up under 2% of the genome in humans, while most sequence regions of the genome is translated into noncoding RNAs (ncRNAs) 3-5. LncRNAs are a novel type of ncRNA longer than 200 nucleotides with no potential to code proteins 6. Numerous studies have demonstrated that lncRNAs may have crucial functions in a great number of biological processes such as tumorigenesis 7-12. For instance, LncRNA GClnc1 could serve as a modular framework for the WDR5 and KAT2A complexes to establish histone modification and promote the carcinogenesis of GC 12. MACC1-AS1 is a LncRNA that catalyzes GC cell metabolic plasticity through the AMPK/Lin28-mediated mRNA steadiness of MACC1 13. LncRNA PINK1-AS promotes tumorigenesis by sponging microRNA-200a in gastric cancer 14. Considering the above, distinguishing lncRNAs associated with GC is necessary to comprehend the progression and develop improved GC treatments.

The objective of our study was to analyze the role of lncRNA PCAT18 in GC. Our results indicated that the lncRNA PCAT18 was downregulated in paired GC tissues and could act as a distinct predictor of overall survival (OS) in GC. Furthermore, mechanistic studies revealed that inhibiting the expression of enhancer of zeste homolog 2 (EZH2) could prevent its linking to the promoter region of PCAT18 and decrease histone3 lysine 27's (H3K27) trimethylation modification in the PCAT18 promoter. Functional studies found that PCAT18, *in vivo* as well as *in vitro*, regulated the cell growth of GC. Further mechanism research revealed that PCAT18 could regulate the expression of p16 by interacting with miR-570a-3p, thus inhibiting cell proliferation of GC. Our research explored the reasons for the activation of PCAT18 and its molecular mechanism. These results imply that the lncRNA PCAT18 may be indicative of a favorable prognosis and provide a theoretical basis for treatment of GC.

## Materials and methods

### Gene Expression Omnibus (GEO) analysis

Microarray gene expression data was retrieved from the GEO dataset. The distinct data sets from GSE79973 were added to the current study. We retrieved the original files, which were standardized by robust multichip average (RMA), from the GEO database. After retrieving the probe sequences from the manufacturers of microarray or GEO, Blast was utilized to re-annotate the probes in GENCODE Release 10 sequence databases for the lncRNAs.

### Tissue collection and ethics statement

In total 60 patients who were subjected to excision of the primary GC at the second hospital of Nanjing for this study between 2014 and 2020. All patients did not receive chemotherapy or radiotherapy before surgery. All collected tissue samples were immersed in RNA Later stabilization solution (Qiagen) and were immediately frozen in liquid nitrogen and stored at -80 °C until RNA isolation. The study was approved by the Ethics Committee of the second hospital of Nanjing (Nanjing, Jiangsu, PR China). And written informed consent from every patient was acquired for this study. Table 1 depicts the clinical features of every patient.

### Cell cultures

Cell lines of human GC were acquired from the Institute of Biochemistry and Cell Biology of the Chinese Academy of Sciences. RPMI 1640 or DMEM with 10% fetal bovine serum (FBS) and 100 U/ml penicillin were used to culture the cells at 37 °C with 5% CO2.

### Cell transfection

Synthetization of the PCAT18 sequence was in accordance to the complete PCAT18 sequence (as based on the NCBI sequence) and then replicated into a pCDNA3.1 vector. X-tremeGENE HP DNA reagent (Roche) was used to transfect the plasmid into cells, and they were processed 48h after the transfection. Typically, cells were seeded at six-well plates and then transfected the next day with specific plasmid (2 ug each well) by using X-tremeGENE HP DNA reagent (6 ul each well), according to the manufacturer's protocol.

### RNA extraction and real-time PCR analysis

TRIzol (Invitrogen) was utilized to separate total RNA from tissues or cells. Then PrimeScript RT Kit (Takara) was utilized to reverse transcribe RNA into cDNA. The SYBR green Premix (Takara) was conducted in order to identify gene expression. The following sequences represent the primers: PCAT18 forward: AGGGATTGGGAGGCCTTTTG, reverse: GTAACGTGACTGGGGTGCAT and GAPDH forward: AGCCACATCGCTCAGACAC, reverse: GCCCAATACGACCAAATCC. P15 forward: GGACTAGTGGAGAAGGTGCG, reverse: GGGCGCTGCCCATCATCATG. P16 forward: CACCGAATAGTTACGGTCGG, reverse: GCACGGGTCGGGTGAGAGTG. P21 forward: GTCCACTGGGCCGAAGAG, reverse: TGCGTTCACAGGTGTTTCTG. P27 forward: TGCAACCGACGATTCTTCTACTCAA, reverse: CAAGCAGTGATGTATCTGATAAACAAGG.

EZH2 forward: TGCACATCCTGACTTCTGTG, reverse: AAGGGCATTCACCAACTCC. The ABI 7500 was used to conduct real-time PCR, and the findings were standardized to GAPDH's expression.

### Cell proliferation assays

To identify cell proliferation MTT assay was used. Transfected cells were transferred to a 96-well plate and determined every 24h. To produce colony formation assays, the transfected cells were transferred to a six-well plate and preserved in the appropriate medium for circa 14 days. Then methanol was used to fixate the colonies, which were thereafter dyed with crystal viole, and lastly the number of dyed colonies were counted.

### EdU assays

A 5-ethynyl-2-deoxyuridine (EdU) detection kit (Ribobio, China) was used, as stated by the manufacturer's instructions, to identify the proliferating cells. Then the cells were observed by fluorescence microscope, and on 5 random chosen fields the ratio of EdU-positive cells were calculated.

### Flow cytometric analysis

Cycle Reagent Kit (BD Biosciences) was used to dye the cells for cell cycle analysis, in accordance to the protocol, andevaluated by FACScan after transfection. The ratio of cells in distinct cell cycle stages, G0/G1, S, and G2/M, were compared and counted.

### Xenograft study

Male athymic BALB/c mice were kept indistinct aseptic surroundings and handled in accordance to the protocols. The SGC7901 cells were transfected with either pCDNA-PCAT18 or an empty vector and collected at a 1 × 10^7^ cells. To suspend the cells, 0.1 ml was injected subcutaneously into one side of the mouse's flank or the other. Every 3 days, tumor's weight and volume were measured in the mice and calculated as width^2^ × length×0.5. After 15 days of injections, the mice were euthanized, and the weight of the tumors s evaluated. The primary tumors were resected, and immunostaining was used to analyze the Ki67 expression. The study was approved by the institutional animal care and use committee of Nanjing Medical University. The animal experiments were performed with the approval of The Institutional Committee for Animal Research and in conformity with national guidelines for the care and the use of laboratory animals.

### Western blot assays

10% SDS-PAGE was used to segregate the protein lysates, then incubated together with distinct antibodies, and lastly densitometry (Bio-Rad) was used to quantify the electrophoretic bands. The EZH2 antibody (1:1000) were acquired from Cell Signaling Technology (Cat#: 5246), and the GAPDH served as control (CST, Cat#: 5174). The p16 antibody was also from Cell Signaling Technology (Cat#: 18769).

### Chromatin immunoprecipitation (ChIP) Assays

The EZ-CHIP KIT was utilized to execute ChIP assays as stated by the instructions of the manufacturer (Millipore). The EZH2 antibody and H3 trimethyl Lys 27 antibody were acquired from Millipore and Abcam, respectively. qPCR was utilized to determine quantification of immunoprecipitated DNA, and the ChIP data was measured in relation to the input DNA: 2^[Input Ct-Target Ct]^ ×0.1×100.

### Luciferase reporter assay

The luciferase assays were performed using a luciferase assay kit (Promega, USA) according to the manufacturer's protocol. Briefly, cells were first transfected with appropriate plasmids in 24-well plates. Next, the cells were collected and lysed for luciferase assay 48 h after transfection. The relative luciferase activity was normalized with renilla luciferase activity.

### RNA immunoprecipitation assays

RNA immunoprecipitation (RIP) experiments were performed using a Magna RIP™ RNA-Binding Protein Immunoprecipitation Kit (Millipore, USA) according to manufacturer's instructions.

### Statistical analysis

SPSS 23 software was used for the statistical analysis. In order to identify the connection between clinical characteristics and PCAT18 expression, Chi-square and* t* tests were conducted. Kaplan-Meier was utilized to evaluate the survival curves and a P value of <0.05 was defined as statistically significant.

## Results

### PCAT18 was downregulated in human GC tissues and was related to poor prognosis

**Figure 1:**
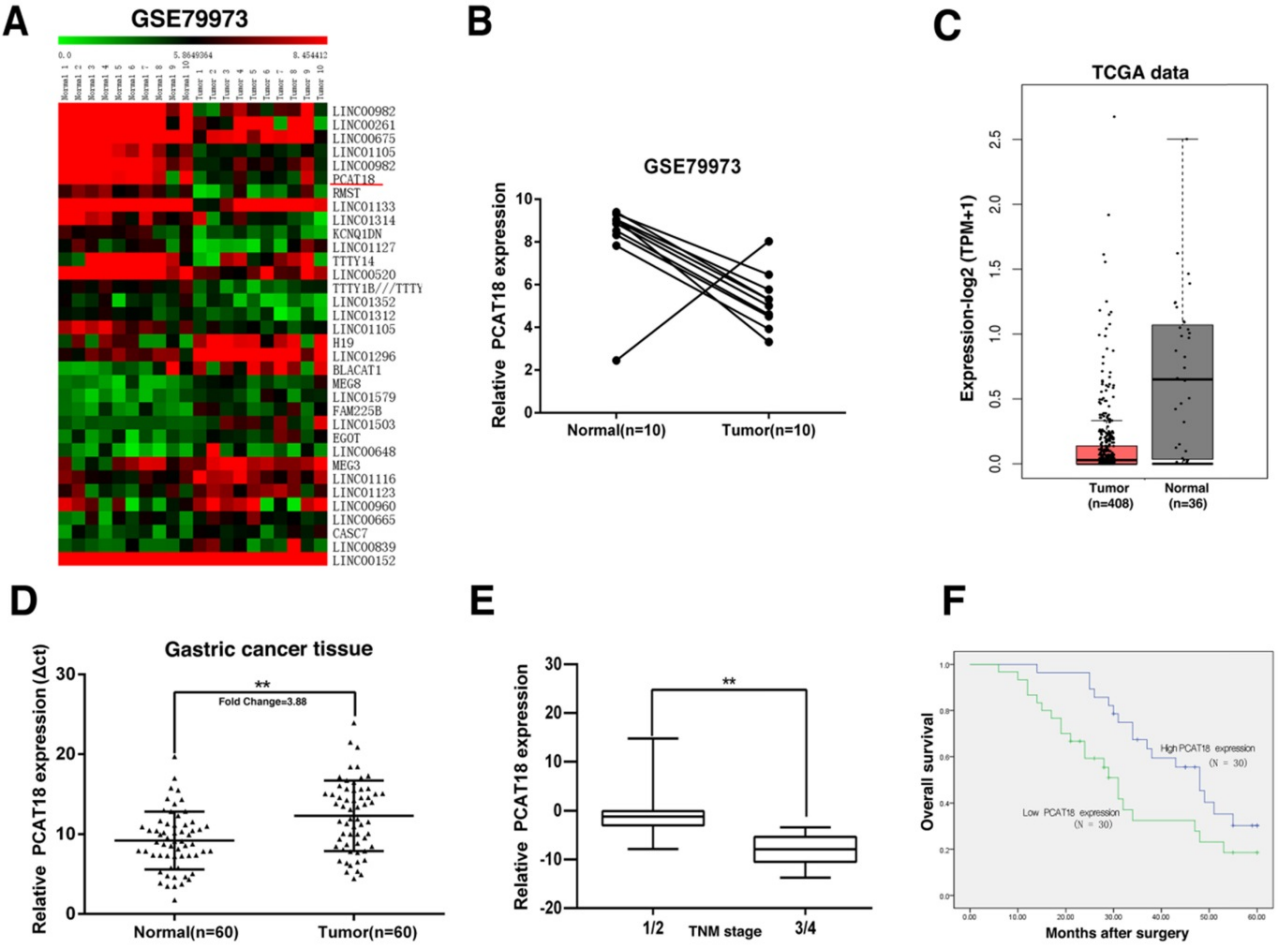
** Screening of PCAT18's expression in GC tissues and clinical characteristics via a bioinformatics analysis. A, B.** Original microarray data, which outlined the lncRNA profiles of ten pairs of human GC tissues and the matching adjoining tumor-free tissues, was retrieved from the GEO Datasets (GSE79973). Then, the standardized signal data was retrieved and -transformed to z-scores. **C.** PCAT18 in RNA-Seq data from TCGA in GC tissues. **D.** Comparison of the proportional expression of PCAT18 in GC tissues (N =60) with the matching tumor-free tissues (N=60). Expression of PCAT18 was determined by qRT-PCR and standardized to GAPDH expression. The findings are represented by the fold-change in the tumor tissues compared to the tumor-free tissues. **E.** A larger quantity of PCAT18 had a positive correlation with the TNM stage.** F.** Compared with patients who had high levels of PCAT18 expression, patients with low levels of PCAT18 expression had decreased survival times. **P<0.01.

Original microarray data, which described the lncRNA profiles of ten paired human GC tissues, was retrieved from the GEO Datasets (GSE79973), to acquire distinctively expressed lncRNA in GC. Then the standardized signal data was transformed to z-scores. As shown in Figure 1A and B, we discovered that PCAT18 was one of the most downregulated lncRNA. RNA-Seq data from TCGA also found that PCAT18 was downregulated in GC tissues (Figure 1C).

To verify the microarray findings, we utilized quantitative real-time PCR (qRT-PCR) to detect PCAT18 expression in 60 paired GC tissues. The expression of PCAT18 was significantly downregulated in 90% (54 of 60) of the GC tissues in contrast to tumor-free tissues (Figure 1D). To evaluate the relation between PCAT18 expression and clinicopathological features, the 60 paired tissues were separated into two different groups according to mean proportion of PCAT18 in GC tissues: a high-PCAT18 group (above the mean, n=30) and a low-PCAT18 group (below the mean, n = 30), and then t-tests were performed. As demonstrated in Table 1, the levels of PCAT18 were also related to the tumor's TNM stages (p=0.004) (Figure 1E). No relationships were found between PCAT18 and clinicopathological characteristic, such as age and sex, etc.

To analyze the relation between PCAT18 expression and GC prognosis, a Kaplan-Meier analysis was conducted to assess the impact of PCAT18 expression on OS. As demonstrated in Figure 1F, the downregulation of PCAT18 indicated a poor prognosis in GC.

### EZH2 could epigenetically inhibit expression of PCAT18 by mediating H3K27me3 histone modification

**Figure 2:**
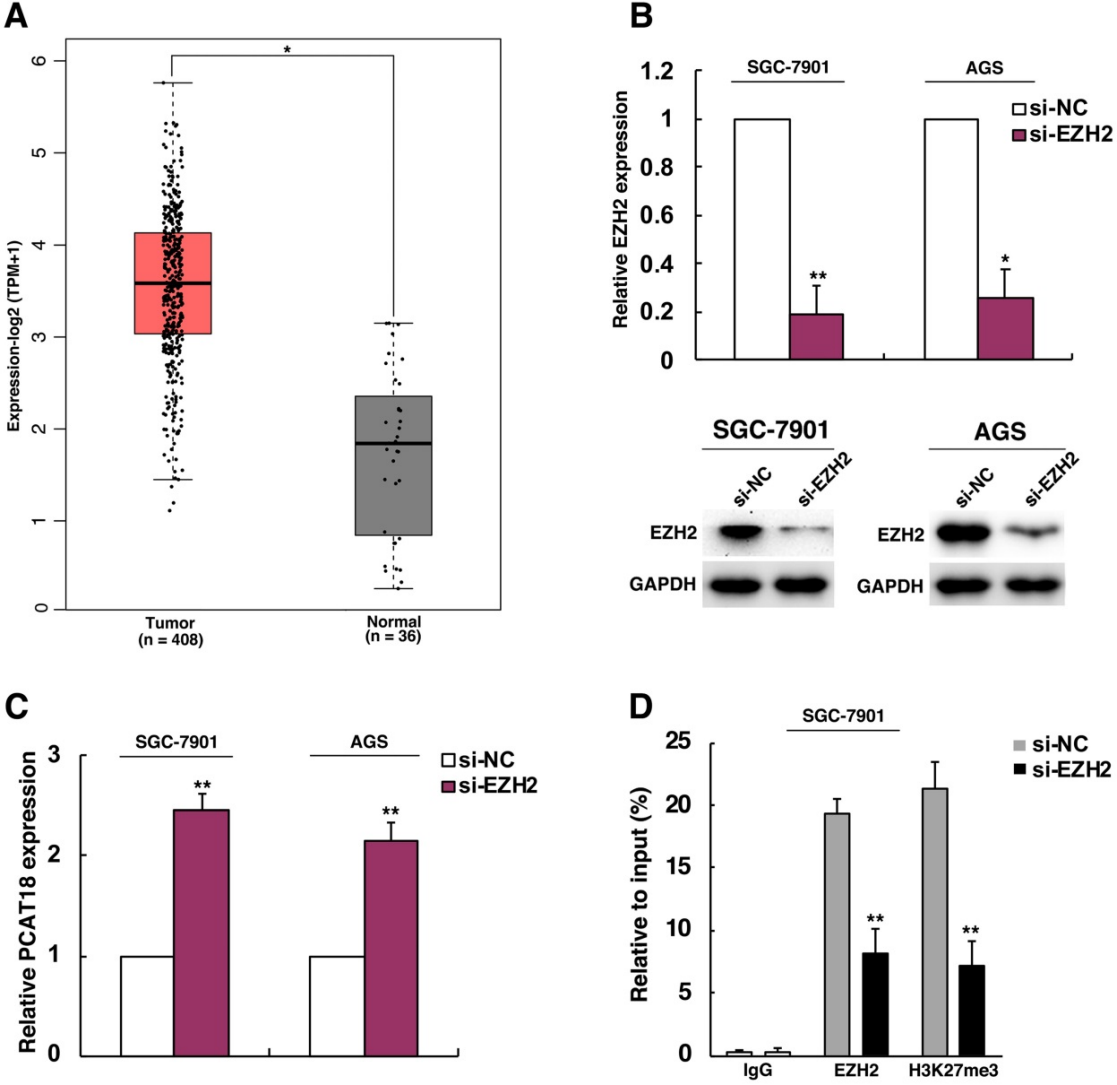
** EZH2 could epigenetically repress PCAT18 expression by H3K27me3 modification. A.** The expression of EZH2 in TCGA data of GC. **B.** Western blot analysis and qPCR of the expression levels of EZH2after cell treatment with si-EZH2 both in SGC7901as well as AGS cells. **C.** The expression of PCAT18 following EZH2's knockdown. **D.** ChIP-qPCR of H3K27me3 and EZH2 of the promoter region of the PCAT18 locus following siRNA treatment aimed at si-EZH2 or si-NC in SGC7901 cells. *P < 0.05, **P < 0.01.

The transcription of lncRNA is typically controlled by epigenetic factors and regulation mediated by transcription factors. For instance, the lncRNA MEG3 was deactivated in tumor following the rise in CpG methylation in the promoter region 15. As an essential part of regulation of methylation, methylation of histone has been known as an important reason for repression and transcriptional activation 16. Then we explored the molecular mechanisms of PCAT18's lower expression in GC. EZH2 is known as a methyltransferase and the main catalytic subunit of the polycomb repressive complex 2 (PRC2), which could catalyze the trimethylation ofH3K27 and generate chromatin compression and transcriptional suppression of target genes. EZH2 could participate in process of cell proliferation, differentiation and tumorigenesis 17-19. Many evidences indicated that EZH2 was activated in many cancer types, including GC 20, 21. Thus, we analyzed if PCAT18 could be an important anti-oncogene, which was EZH2's target of inhibition in GC. As visualized in Figure 2A, EZH2 was upregulated in TCGA data of GC. To confirm the presumed role of EZH2 in PCAT18 suppression, firstly, EZH2's expression in SGC7901 and AGS cells was knocked down. Then we found that knockdown of EZH2 could induce PCAT18 expression (Figures 2B and 2C). For additional verification of the epigenetic suppression of PCAT18 by EZH2, we conducted ChIP assays to determine if EZH2 and H3K27me3 could take up the promoter of PCAT18 in GC cells after EZH2's knockdown, while IgG antibody served as a negative control. The ChIP-qPCR data revealed that the PCAT18 promoter was enhanced by EZH2 and H3K27me3, and EZH2's knockdown could reduce the binding of EZH2 and modification of H3K27me3 in the PCAT18 promoter (Figure 2D).

### PCAT18 suppresses cell growth of GC *in vitro*

**Figure 3:**
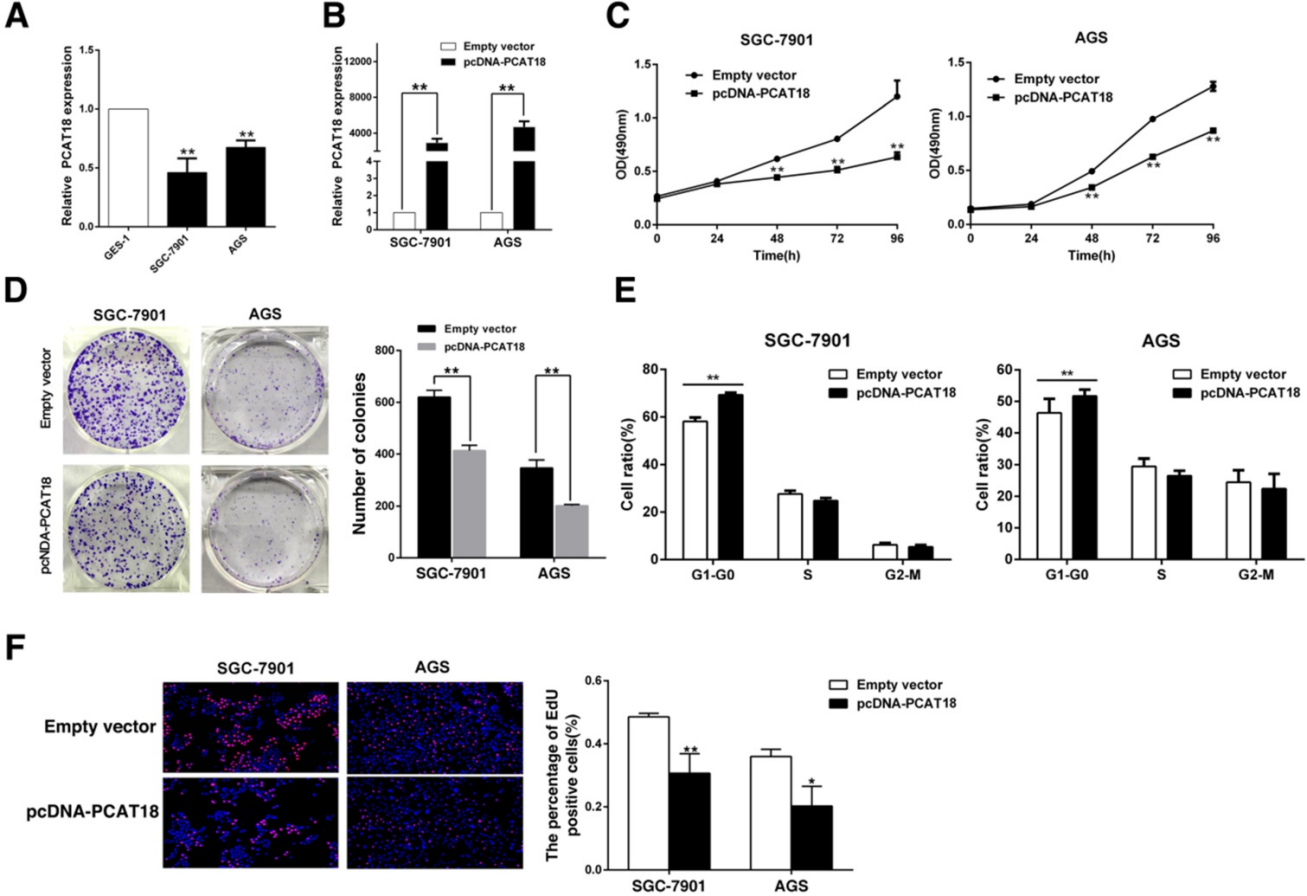
** PCAT18 inhibits GC cell growth *in vitro*. A.** qRT-PCR analysis of PCAT18 expression in tumor-free GES1 and GC cells. **B.** qPCR was utilized to test the proportional expression level of PCAT18 in the SGC7901 and AGS cells transfected with pcDNA-PCAT18. **C, D.** To identify the sustainability of the GC cells transfected with pcDNA-PCAT18, MTT assays were done in triplicate. To identify the growth of the GC cells transfected with pcDNA-PCAT18 colony formation assays were conducted, and then the colonies were captured and calculated. **E.** Flow cytometry was used to analyze the cell cycle 48 h after transfection. The bar chart visualizes the ratio of cells in the G1-G0, S, or G2-M phase, as noted. **F.** Proliferating SGC7901 and AGS cells werestained with red EdU staining and the cell nuclei were marked with blue DAPI. The results of the independent experiments in triplicate are shown in representative data and images. *P < 0.05, **P < 0.01.

To determine the biological function of PCAT18 in GC, qRT-PCR was used to analyze PCAT18 expression in human GC cell lines (SGC7901, AGS) and gastric epithelium cell line (GES-1). As visualized in Figure 3A, the GC cell lines expressed lower levels of PCAT18 than GES-1. Next, we manipulated the level of PCAT18 in the GC cell lines via transfection with PCAT18 plasmids (Figure 3B). Following transfection, the MTT assay demonstrated that exogenous PCAT18 could significantly suppress cell growth in SGC7901 and AGS cell lines in contrast to the control cells (Figure 3C). Likewise, colony formation assays showed that following overexpression of PCAT18 in the SGC7901 and AGS cells, clonogenic viability was significantly reduced (Figure 3D). Next, flow cytometry was used. As shown in Figure 3E, for the SGC7901 and AGS cells, the cell cycle progression of G1-G0 phase is significantly stagnated after overexpression of PCAT18. EdU immunostaining confirmed that the enhanced expression of PCAT18 could significantly decrease the ratio of growing cells (Figure 3F).

### PCAT18 could inhibit cell proliferation of GC by inducing p16 expression

**Figure 4:**
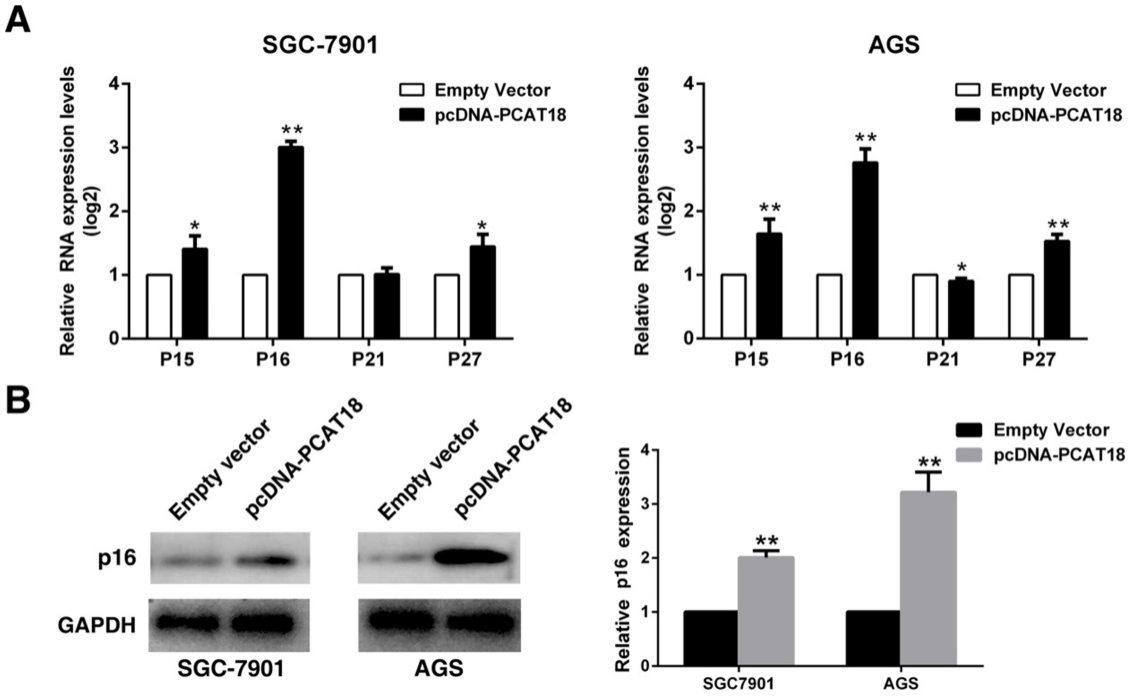
** Overexpression of PCAT18 could inhibit expression of P16 in GC cells. A and B.** The expression of p15, p16, p21 and p27 following the upregulation of PCAT18 was identified by qRT-PCR and WB.

To explore whether PCAT18 could influence G0/G1 arrest, we analyzed the expression of Cdk inhibitors (CKIs). CKIs closely regulates the kinase activity of Cdk/cyclin complexes, which could act as barriers to obstruct the cell cycle's development. Cyclin-dependent kinases (CDK) are serine/threonine kinases, and CKIs regulate their catalytic activities. The expression of P15, P16, P21 and P27 was detected in the AGS and 7901 cells following overexpression of PCAT18, and it revealed that the P16's expression level was significantly enhanced (Figure 4A and 4B).

### PCAT18 suppresses tumorigenesis of GC cells *in vivo*

**Figure 5:**
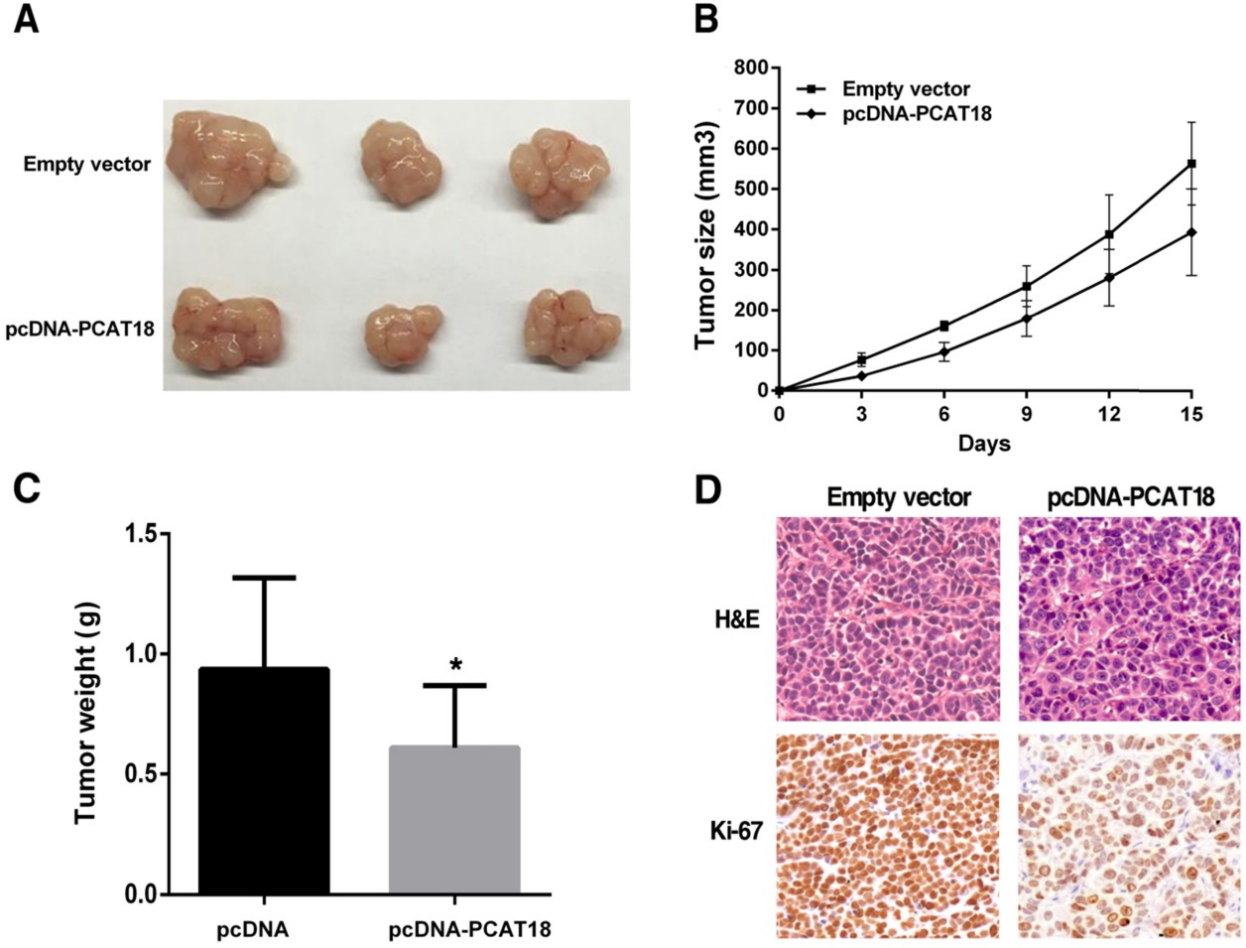
** The influence of PCAT18 on tumorigenesis *in vivo*. A, B.** pcDNA-PCAT18 or empty vector was first transfected into the 7901 cells, before injecting into the nude mice (n=3). Each two days after the injection, the tumor volumes were measured. The bars represent the SD. **C.** The weight of the tumors is depicted as mean ± SD (n=3). **D.** The tumor portions underwent IHC and H&E staining by an antibody opposing Ki-67. *P < 0.05.

To analyze that PCAT18 could also affect tumorigenesis *in vivo*, we collected SGC7901 cells, which were transfected with the pCDNA-PCAT18 and control vector and injected them into the mice. As shown in Figure 5A and 5B, development of the tumor was significantly delayed in the overexpression PCAT18 group in contrast to the control group. As shown in Figure 5C, the tumor's average weight was less in the pcDNA-PCAT18 group compared to the control group. Using immunohistochemical staining, we found that after transfection with pcDNA-PCAT18, Ki-67's proliferation index was significantly reduced (Figure 5D). In sum, these results showed that the overexpression of PCAT18 could inhibit GC cell proliferation *in vivo*.

### PCAT18 could regulate the expression of p16 by interacting with miR-570a-3p, thus inhibiting cell proliferation of GC

**Figure 6:**
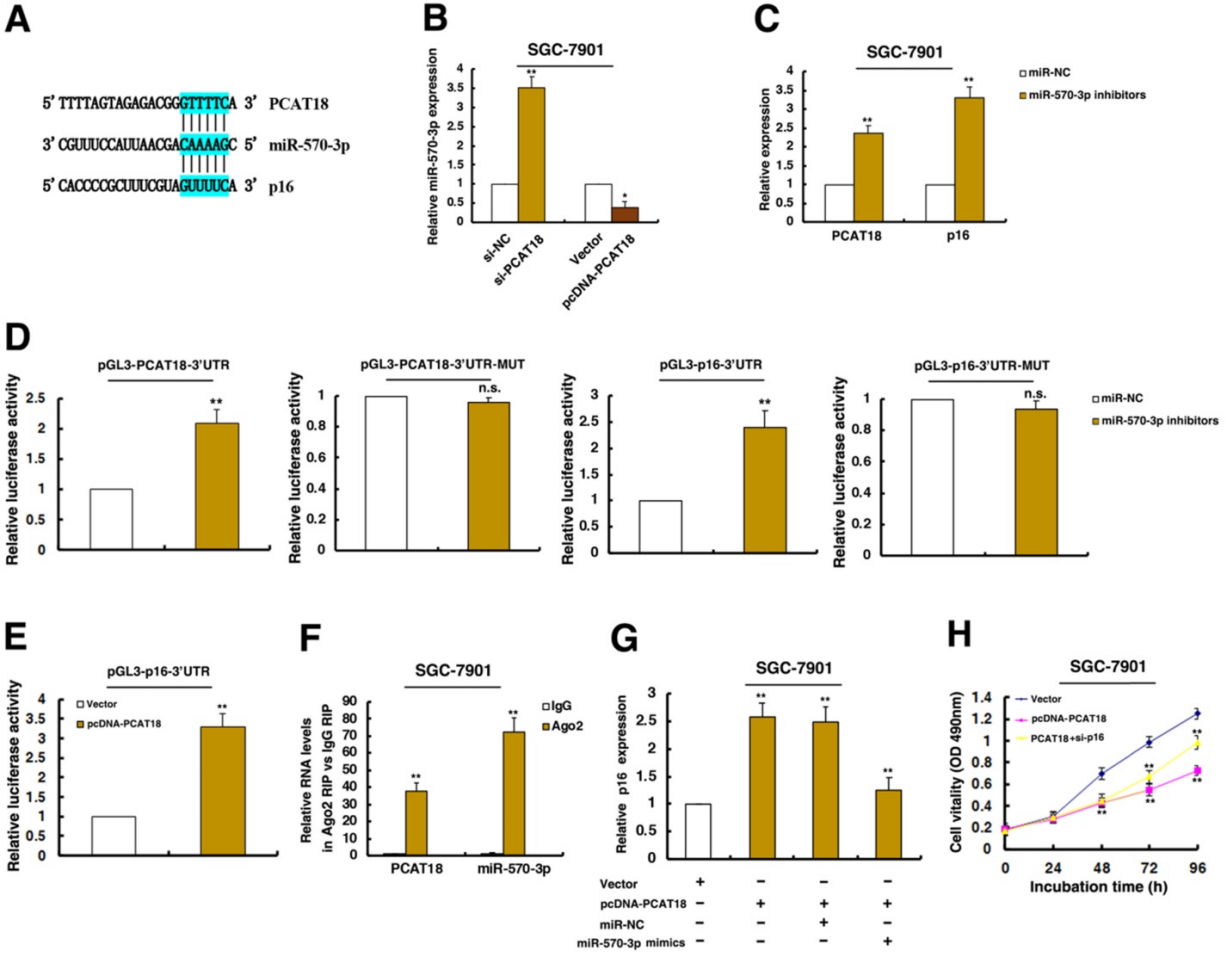
** The PCAT18 could regulate the expression of p16 by interacting with miR-570a-3p, thus inhibiting cell proliferation of GC. A.** The RNAup algorithm predicted potential binding of miR-570a-3p to PCAT18 and to p16, with considerable sequence complementary in the indicated regions. **B and C.** qRT-PCR assays detected the expression of miR-570a-3p after knockdown of PCAT18 and overexpression of PCAT18 in SGC-7901 cells. qRT-PCR assays detected the expression of PCAT18 and p16 after inhibition of miR-570a-3p. **D.** The 3´UTR of p16 mRNA and full length of PCAT18 were respectively inserted downstream of a luciferase gene. The reporter vector was co-transfected with a renilla luciferase vector (for normalization), which were treated by miR-570a-3p inhibitors or control. The luciferase signals of both reporter genes were significantly increased when cells were treated with miR-570a-3p inhibitors. Mutant 3´UTR of p16 and PCAT18 is not affected by miR-570a-3p. **E.** The luciferase signal of reporter gene was increased when overexpression of PCAT18. **F.** RIPs experiments showed that PCAT18 and miR-570a-3p were obviously enriched in Ago2-immunoprecipitation relative to control IgG. **G and H.** The promotion of p16 by PCAT18 was significantly reversed by miR-570a-3p mimics, by using qPCR assays. MTT assays showed that knockdown of p16 could reverse PCAT18-mediated growth inhibition. *P < 0.05, **P < 0.01.

Recently, a new regulatory mechanism has been identified in which crosstalk between lncRNAs and mRNAs occurs by competing for shared microRNAs (miRNAs) response elements. Interestingly, a microRNA, miR-570a-3p, was predicted to target both PCAT18 and p16 (Figure 6A). Further experiments found that knockdown PCAT18 increased miR-570a-3p expression, and opposite results were found in PCAT18 overexpression (Figure 6B). Moreover, treatment by miR-570a-3p inhibitors significantly induced PCAT18 and p16 levels (Figure 6C).

To validate the effects of miR-570a-3p, we cloned the 3'UTR of PCAT18 and mutant 3'UTR of PCAT18 downstream of luciferase genes and co-transfected these reporters with miR-570a-3p inhibitors in GC cells. As expected, miR-570a-3p significantly increased the luciferase signals of reporters of 3'UTR of PCAT18 (Figure 6D). However, miR-570a-3p had no effect on mutant reporters of 3'UTR of PCAT18 (Figure 6D). And we also cloned the 3'UTR of p16 and mutant 3'UTR of p16 downstream of luciferase genes and co-transfected these reporters with miR-570a-3p inhibitors in GC cells. As shown in Figure 6D, miR-570a-3p obviously increased the luciferase signals of reporters of 3'UTR of p16. And miR-570a-3p had no effect on mutant reporters of 3'UTR of p16 (Figure 6D). These data directly confirmed that miR-570a-3p could target PCAT18 and p16. Importantly, overexpression of PCAT18 significantly induced the luciferase intensity of 3'UTR of p16, indicating that PCAT18 is required for the post-transcriptional expression of p16 (Figure 6E).

miRNAs are known to be present in the cytoplasm in the form of miRNA ribonucleoprotein complexes (miRNPs) that also contain Ago2, the core component of the RNA-induced silencing complex (RISC). To test whether PCAT18 associates with Ago2, RIP assays were performed in GC cells extracts using antibodies against Ago2. As shown in Figure 6F, PCAT18 and miR-570a-3p were all enriched in Ago2-immunoprecipitation relative to control IgG.

Furthermore, the promotion of p16 by PCAT18 was reversed when treatment by miR-570a-3p mimics (Figure 6G). Moreover, knockdown of p16 could reverse PCAT18-mediated growth inhibition (Figure 6H). All these results prove the relationship between PCAT18/miR-570a-3p/p16 in tumorigenesis of GC.

## Discussion

Up to now, is has been confirmed that the discovered lncRNAs are important elements of human diseases. During the current study, we discovered that PCAT18 was significantly lower in GC tissues in contrast to non-tumor tissues. There was also a favorable association between lower expression of PCAT18 and the TNM stage. In addition, lower PCAT18 expression was correlated to a poor prognosis and could serve as an independent predictor of survival in GC. These data indicated that PCAT18 could be an important part of the tumorigenesis of GC. The atypical expression of lncRNAs may be part of various tumorigeneses and could serve as an indicator of prognosis. Our studies also found that the lncRNAs HOXC-AS3 and TINCR could be used as prognostic factors of GC 11, 22.

Anti-oncogenes are generally inhibited through epigenetic modifications in cancer cells. Prior studies have revealed that epigenetic regulators like DNA methylation or histone modification, could regulate expression of tumor suppressor genes 23. For instance, the lncRNA MEG3 was deactivated in cancers, because of a rise in CpG DNA methylation in MEG3's promoter 15.

EZH2 could function as a histone methyltransferase which could specifically induce H3K27me3 modifications to the promoter of targeted genes 24. Activation of EZH2 has been found in many types of tumors. And EZH2 could regulate cell migration and growth in tumorigenesis 25. Here we established that the EZH2 were upregulated in GC tissues. And knockdown of EZH2 could significantly upregulate expression of PCAT18. Then ChIP assays found that suppression of EZH2 could block its binding and decrease H3K27me3 modification in the promoter region of PCAT18. Our data indicated that lower expression of PCAT18 was partially mediated by histone modification.

Previous studies indicated that in metastatic prostate cancer, PCAT18 is strongly expressed and that knockdown of PCAT18 obviously suppressed proliferation and migration of the prostate cancer cells 26. Nevertheless, the function role and mechanism of PCAT18 in GC still needs to be clarified. In our study, we discovered that PCAT18 inhibited the cell proliferation in GC *in vitro* as well as *in vivo*. In addition, the overexpression of PCAT18 induced evident G0/G1 inhibition. In human cancers, the deactivation of CKIs could cause disorders in the cell cycle and promote cell proliferation in cancer, including p15, p16, p21, p27, etc. 27. The inhibitory functional roles of CKI have been clearly explained in tumorigenesis. Our results indicated PCAT18 may be an anti-oncogene in GC, partly through affecting p16 expression. Mechanism research findings revealed that PCAT18 could regulate the expression of p16 by interacting with miR-570a-3p, thus inhibiting cell proliferation of GC by modulating cell cycle. And many studies have confirmed that lncRNA interacts with miRNA to promote tumorigenesis 28. In addition, plenty studies have indicated that lncRNAs could manage the cell cycle 28. Our current study detected a new histone modification-mediated epigenetic suppression of PCAT18 and lncRNA/miRNA-mediated interaction regulator of cell proliferation and cell cycle in GC. Our results may offer an approach for targeting PCAT18 as a possible biomarker in GC.

## Figures and Tables

**Table 1:** The clinic-pathological factors of 60 GC patients

Characteristics	(%) of patients	Expression of PCAT18		P-value
		low	high	
**Sex**				0.118
male	34 (57%)	14	20	
female	26 (43%)	16	10	
**Age**				0.196
≤60	31 (52%)	18	13	
>60	29 (48%)	12	17	
**Histological subtype**				0.121
Squamous cell carcinoma	28 (47%)	11	17	
Adenocarcinoma	32 (53%)	19	13	
**TNM stage**				0.004**
I and II	29 (52%)	9	20	
III and IV	31 (48%)	21	10	
**Lymph node metastasis**				0.436
negative	33 (55%)	15	18	
positive	27 (45%)	15	12	
**Tumor size**				0.194
≤3 cm	27 (28%)	11	16	
>3 cm	33 (72%)	19	14	

** p<0.01.
